# Comparative Analysis and Phylogenetic Study of *Dawkinsia filamentosa* and *Pethia nigrofasciata* Mitochondrial Genomes

**DOI:** 10.3390/ijms25053004

**Published:** 2024-03-05

**Authors:** Cheng-He Sun, Chang-Hu Lu

**Affiliations:** College of Life Sciences, Nanjing Forestry University, Nanjing 210037, China; sunchenghe@njfu.edu.cn

**Keywords:** Smiliogastrinae, mitochondrial genome, sequence analysis, phylogenetic evolution

## Abstract

Smiliogastrinae are recognized for their high nutritional and ornamental value. In this study, we employed high-throughput sequencing technology to acquire the complete mitochondrial genome sequences of *Dawkinsia filamentosa* and *Pethia nigrofasciata*. The gene composition and arrangement order in these species were similar to those of typical vertebrates, comprising 13 protein-coding genes, 22 tRNA genes, 2 rRNA genes, and 1 non-coding region. The mitochondrial genomes of *D. filamentosa* and *P. nigrofasciata* measure 16,598 and 16,948 bp, respectively. Both *D. filamentosa* and *P. nigrofasciata* exhibit a significant preference for AT bases and an anti-G bias. Notably, the AT and GC skew values of the ND6 gene fluctuated markedly, suggesting that the selection and mutation pressures on this gene may differ from those affecting other genes. Phylogenetic analysis, based on the complete mitochondrial genomes of 23 Cyprinidae fishes, revealed that *D. filamentosa* is closely related to the sister group comprising *Dawkinsia denisonii* and *Sahyadria chalakkudiensis.* Similarly, *P. nigrofasciata* forms a sister group with *Pethia ticto* and *Pethia stoliczkana*.

## 1. Introduction

Tan and Armbruster [1] reclassified *Puntius* and related species under the subfamily Smiliogastrinae, following a phylogenetic analysis of fish genera within the order Cypriniformes. At present, there are 25 genera in the subfamily Smiliogastrinae according to the National Center for Biotechnology Information, including 9 single-species genera. In Bangladesh, extensive research has been conducted on the abundance and distribution of barbs in various habitats, including rivers, floodplains, and mountain streams [2,3]. Most species of the Smiliogastrinae subfamily have bright colors, and accurate identification and monitoring of these fish species are crucial, given their important economic and ornamental value.

Mitochondrial DNA (mtDNA) is a compact genome, typically 15–20 kb in size, and exists as a closed loop separate from the nuclear genome. mtDNA exhibits properties such as maternal inheritance, simple structure, autonomous replication, high mutation rate, and consistent mutation probability, making it an invaluable tool in studying species evolution, phylogenetic relationships, and population genetics. mtDNA has been used to examine genetic differentiation within and between closely related species [4,5,6]. Recent advancements in DNA sequencing technology have greatly facilitated the rapid and accurate acquisition of fish mitochondrial genome data. Moreover, several complete fish mtDNA sequences have been reported, enriching our understanding of classification and systematic evolution within this group. Thus far, complete mitochondrial genome sequences have been reported for only 19 species of Smiliogastrinae [7].

In this study, we employed high-throughput sequencing technology to determine and analyze the complete mitochondrial genome sequences of *Dawkinsia filamentosa* and *Pethia nigrofasciata*. By comparing these genomes with those of 19 related species, we aimed to elucidate the molecular phylogenetic relationships within this group. These findings are expected to contribute fundamental insights for the germplasm identification and genetic diversity conservation of Smiliogastrinae.

## 2. Results

### 2.1. Mitochondrial Genome Analysis

**Figure 1:**
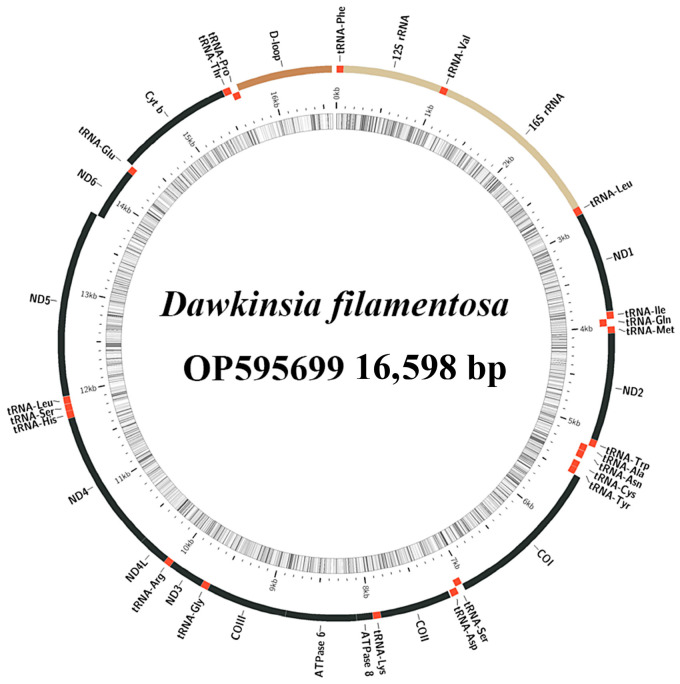
Structure of *Dawkinsia filamentosa* mitochondrial genome.

**Figure 2:**
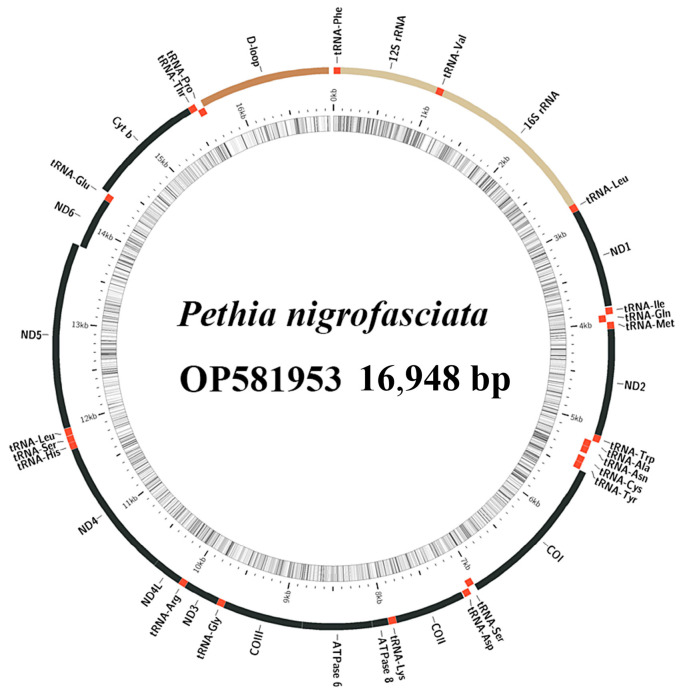
Structure of *Pethia nigrofasciata* mitochondrial genome.

The total mitochondrial genome lengths of *D. filamentosa* and *P. nigrofasciata* are 16,598 and 16,948 bp, respectively (Figure 1 and Figure 2). In *D. filamentosa*, the nucleotide compositions are 33.9, 24.2, 27.3, and 14.7%, for A, T, G, and C, respectively; whereas in *P. nigrofasciata* the nucleotide compositions are 33.2, 26.7, 24.9, and 15.1%, for A, T, G, and C, respectively. Both species exhibit high AT content in their mitochondrial genomes, which comprise 37 genes (13 protein-coding genes [PCGs], 22 tRNAs, and 2 rRNAs), and one non-coding control region (Table 1 and Table 2). Seven gene overlaps were found in both species, with the longest overlap (7 bp) between ATP8 and ATP6, and ND4L and ND4.

In the mitochondrial genomes of both species, the heavy and light strands encode a different number of genes. The heavy strand encodes 28 genes, including 12 PCGs, 14 tRNAs, and 2 rRNAs; the light strand encodes 9 genes, comprising 1 PCG and 8 tRNAs. The non-coding control regions of *D. filamentosa,* located between tRNA-Pro and tRNA-Phe, span 931 bp, and the nucleotide compositions of A, T, G, and C are 36.2, 33.0, 11.8, and 19.0%, respectively. The non-coding control area of *P. nigrofasciata* is similarly positioned with a length of 1288 bp, and the A, T, G, and C compositions are 36.7, 33.4, 11.5, and 18.4%, respectively. The overall AT content of the *D. filamentosa* mitochondrial genome is 58.1%, with AT skew and GC skew values of 0.166 and −0.300, respectively (Table 3); for *P. nigrofasciata*, these values are 59.9%, 0.108, and −0.245, respectively (Table 4).

### 2.2. Protein-Coding Gene Analysis

Among the 13 PCGs in *D. filamentosa*, only *COI* uses GTG as the starting codon, while the remaining 12 PCGs initiate with ATG. *COII*, *ND2*, *ND3*, and *ND4* have incomplete termination codons (T), *ATP6* and *COIII* terminate with TA-, and the remaining seven PCGs end with the complete termination codons TAA or TAG. Among the 13 PCGs of *P. nigrofasciata*, only *COI* starts with GTG, while the remaining 12 PCGs begin with ATG. *COII*, *COIII*, *Cytb*, *ND2*, *ND3*, and *ND4* have incomplete termination codons (T), *ATP6* ends with TA-, and the remaining six PCGs terminate with the complete termination codons TAA or TAG.

A preliminary analysis of the relative synonymous codon usage (RSCU) and amino acid composition of the 13 PCGs in the mitochondrial genomes of *D. filamentosa* and *P. nigrofasciata* was performed. The results showed that leucine (Leu) was the most frequently used amino acid, constituting 12.69 and 11.25% of the 3798 and 3800 amino acids encoded, respectively. This was followed by alanine (Ala, 8.23 and 8.8%, respectively) and threonine (Thr, 8.84 and 8.22%, respectively). Cysteine (Cys) was the least abundant, accounting for only 0.66% (Table 5 and Table 6). *Dawkinsia filamentosa* and *P. nigrofasciata* showed a preference for 25 and 26 codons (RSCU ≥ 1) in their 13 PCGs, respectively.

### 2.3. tRNAs and rRNAs Gene Analysis

The 22 tRNA genes in *D. filamentosa* are 67–77 bp long. The longest gene, Leu2-tRNA, is 74 bp long, while the shortest, Cys-tRNA, is 67 bp (Table 1). In *P. nigrofasciata*, the sequence length of the 22 tRNAs genes is 67–77 bp, with Lys-tRNA being the longest at 77 bp, and Cys-tRNA being the shortest at 67 bp (Table 2). Of the 24 RNA genes of *D. filamentosa* and *P. nigrofasciata*, 8 are located on the L chain and 16 on the H chain.

The total length of the 16S rRNA gene in the mitochondrial genome of *D. filamentosa* is 1695 bp (Table 3), with an AT content of 57.4%. The 12S rRNA measures 955 bp (Table 3), with an AT content of 51.8%. In *P. nigrofasciata*, the 16S rRNA gene is 1684 bp in length (Table 4), with an AT content of 59.1%, and the 12S rRNA gene is 956 bp (Table 4), with an AT content of 51.7%.

### 2.4. Phylogenetic Analysis

**Figure 3:**
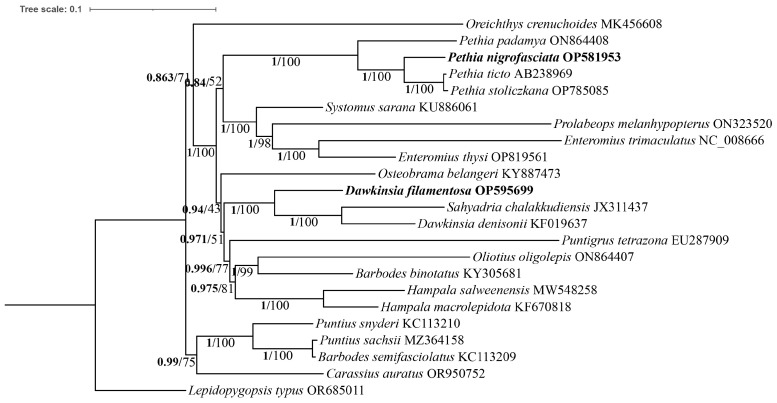
Phylogenetic tree inferred from the nucleotide sequences of 13 protein-coding genes. Numbers at nodes represent the posterior probability values for Bayesian analysis and bootstrap values for maximum likelihood analysis.

Using the mitochondrial genomes of 23 Cyprinidae fish, 13 PCG tandem sequences were used to construct Bayesian inference (BI) and maximum likelihood (ML) phylogenetic trees (Figure 3). The trees consistently supported the monophyly of *Enteromius*, *Hampala*, and *Pethia*. However, further research is required regarding the monophyletic relationships of the other three genera: *Barbodes*, *Dawkinsia*, and *Puntius*. Notably, both phylogenetic trees strongly support *D. filamentosa* forming a sister group with *Dawkinsia denisonii* and *Sahyadria chalakkudiensis* (BS = 100, PP = 1). Similarly, *P. nigrofasciata* is strongly indicated to be a sister group of *Pethia ticto* and *Pethia stoliczkana* (BS = 100, PP = 1).

## 3. Discussion

We used high-throughput sequencing technology to obtain the complete mitochondrial genome sequences of *D. filamentosa* and *P. nigrofasciata,* measuring 16,598 and 16,948 bp, respectively. The structural characteristics of these sequences align with those of previously published studies of Smiliogastrinae fish, underscoring the high evolutionary conservation of the mitochondrial genome in this group [8]. AT and GC skew values are indicative of base content differences between the heavy and light chains, and larger absolute values indicate more significant differences in the base composition between the two [9]. *Dawkinsia filamentosa* and *P. nigrofasciata* mitochondrial DNA sequences exhibit a higher AT content and a lower GC content, thus exhibiting a clear AT preference. This preference has also been observed in other Smiliogastrinae species, but the content varies slightly depending on the species. This base preference may be related to natural mutations and selection pressure during replication and transcription. Unless there are significant changes in the number of coding regions and tRNA bases, each gene is similar to other species of Smiliogastrinae and has a high homology. Typical features of AT duplication also exist in non-coding regions [10]. The base distribution in PCGs was relatively uniform, except for the first codon, whereas both the second and third codons exhibited significant anti-G bias. This uneven distribution is likely due to limitations imposed by amino acid compositions and differences in codon usage frequencies [11].

Leucine, a hydrophobic amino acid, was the most frequently used in the 20 amino acid-encoding proteins, a trend consistent with 19 other fish species of Smiliogastrinae [8]. This may be related to the composition of transmembrane proteins encoded by mitochondrial genes. Notably, the absolute value of AT skew is the highest in the base composition of the *ND6* gene, and the GC skew value is the only positive value, with significant fluctuations in AT/GC bias values. The proton-transporting NADH-quinone oxidoreductase, also known as complex I, is a multi-subunit membrane protein complex that catalyzes electron transfer in the NADH respiratory chain and provides approximately 40% of the proton power required for ATP synthesis in vertebrates [12,13]. The ND6 subunit, a critical component of complex I, suggests differential selection and mutation pressures related to respiratory metabolism compared to other genes.

The total lengths of the two rRNA genes of *D. filamentosa* and *P. nigrofasciata* were 2650 and 2640 bp, respectively. The intervals between the 12S rRNA and 16S rRNA gene were consistent with Val-tRNA, aligning with the typical characteristics of Smiliogastrinae. Given its stability, the 12S rRNA is often used for fish identification and phylogenetic studies [14]. Studies have shown that base mismatches are also commonly present in the secondary structure of tRNA, as they help to mitigate harmful mutations in the non-recombinant mitochondrial genome [15]. The mitochondrial control region, containing elements regulating mitochondrial genome replication and gene expression, offers insights into DNA replication, transcription mechanisms, and evolutionary laws. The significant difference in D-loop length variance as it was observed in our work was also reported previously in the investigated subfamily Smiliogastrinae [7].

Most current phylogenetic studies of Smiliogastrinae rely on individual genes [16,17]. However, in our study we used concatenated sequences of 13 PCGs to construct partial Smiliogastrinae phylogenetic trees using ML and BI methods. These analyses confirmed the monophyletic relationships of *Enteromius*, *Hampala*, and *Pethia*, while suggesting the need for further research on the monophyly of *Barbodes*, *Dawkinsia*, and *Puntius*. In addition, both analyses identified *D. filamentosa* (comprising *Dawkinsia denisonii* and *Sahyadria chalakkudiensis*) and *P. nigrofasciata* (alongside *Pethia ticto* and *Pethia stoliczkana*) as sister groups.

In summary, this study is the first to analyze the mitochondrial DNA genome structure of *D. filamentosa* and *P. nigrofasciata* and to explore their phylogenetic relationships based on multi-gene tandem sequences. These results provide new insights and reference points for further research into the species evolution, classification, identification, and diversity of Smiliogastrinae.

## 4. Materials and Methods

### 4.1. Experimental Materials and DNA Extraction

In September 2021, fish samples were collected from the Huadiwan Flower, Bird, Fish, and Insect Wholesale Market in Liwan District, Guangzhou City, Guangdong Province (23°3′48″ N, 113°12′18″ E). Following preliminary morphological identification, fresh muscle tissue from the back and abdomen were excised and stored in 1.5 mL Eppendorf centrifuge tubes. These samples were soaked in 95% alcohol for 48 h and then preserved at −20 °C. Genomic DNA was extracted using the traditional chloroform tris-saturated phenol method [18]. The concentration of genomic DNA was measured with a NanoDrop ultra-micro spectrophotometer, and DNA integrity was verified by 1% agarose gel electrophoresis.

### 4.2. High-Throughput Sequencing and Gene Annotation

DNA samples that passed the quality inspection were fragmented to approximately 200 bp using a Covaris ultrasonic crusher (Covaris, Woburn, MA, USA) for small genomic DNA library construction. The HiSeq 2500 high-throughput sequencing platform was used for sequencing (Guangzhou Tianyi Huiyuan Gene Technology Co., Ltd., Guangzhou, China). SOAPdenovo 2.04 software (http://soap.genomics.org.cn/soapdenovo.html, accessed on 25 January 2024) [19] was used to assemble clean reads and optimize local assembly based on read parings and overlap with default parameters; gaps introduced during scaffold splicing were compensated and repaired using GapCloser 1.12 software (http://soap.genomics.org.cn/soapdenovo.html, accessed on 25 January 2024) [20], with a redundant segment subsequently removed to obtain the final assembly result. The mitochondrial genome was extracted and assembled using NOVOPlasty v4.3.1 software [21]. The assembled complete mitochondrial genome sequence was annotated on the MITOS web server (http://mitofish.aori.u-tokyo.ac.jp/, accessed on 25 January 2024), involving PCGs, RNA, and non-coding regions. This was supplemented by manual comparison and correction to precisely determine the position and length of each gene. MitoAnnotator (https://mitofish.aori.u-tokyo.ac.jp/annotation/input/, accessed on 25 January 2024) was used to create mitochondrial genome structure maps for *D. filamentosa* and *P. nigrofasciata*.

### 4.3. Data Analysis

The tRNA genes were predicted using the online tool tRNAscan-SE (http://trna. ucsc. edu/tRNAscan-SE/, accessed on 25 January 2024) [22]. Base composition and codon usage were analyzed, and AT and GC skew values were calculated using MEGA-X software [23]. We retrieved and downloaded the complete mitochondrial genome sequences of 19 Smiliogastrinae fish species from GenBank (https://www.ncbi.nlm.nih.gov/, accessed on 25 January 2024; Table 7), using *Lepidopygopsis typus* (OR685011) [24] as outgroup. Phylogenetic trees of Smiliogastrinae fish were constructed based on the tandem sequences of 13 PCGs, using the maximum likelihood [25] and Bayesian inference [26] methods, respectively.

Multiple sequence alignment was performed using MAFFT v7.313 [27]. The optimal partition model was determined using ModelFinder [28], based on the Akaike Information Criterion [29]. An ML tree was constructed with IQ-TREE [30] under the edge-linked partition model for 50,000 ultrafast bootstraps, as well as the Shimodaira–Hasegawa-like approximate likelihood-ratio test. To construct the BI tree, the following steps were conducted. The aligned FASTA file was converted into a NEXUS file and imported into CIPRES software (https://www.phylo.org/, accessed on 25 January 2024) under the partition model (two parallel runs, 100,000,000 generations), in which the initial 25% of sampled data were discarded as burn-in [31]. The phylogenetic tree was then edited using FigTree v1.4.4 (http://tree.bio.ed.ac.uk/software/figtree/, accessed on 25 January 2024).

## Figures and Tables

**Table 1:** Annotation of the mitochondrial genome of *Dawkinsia filamentosa*.

Gene	Position		Size	Intergenic Nucleotides	Codon		Strand
	From	To			Start	Stop	
tRNA-Phe	1	69	69	0			H
12S rRNA	70	1024	955	0			H
tRNA-Val	1025	1096	72	0			H
16S rRNA	1097	2791	1695	0			H
tRNA-Leu2	2792	2868	77	0			H
ND1	2870	3844	975	1	ATG	TAA	H
tRNA-Ile	3849	3920	72	4			H
tRNA-Gln	3919	3989	71	−2			L
tRNA-Met	3992	4061	70	2			H
ND2	4062	5106	1045	0	ATG	T	H
tRNA-Trp	5107	5177	71	0			H
tRNA-Ala	5179	5247	69	1			L
tRNA-Asn	5249	5321	73	1			L
tRNA-Cys	5357	5423	67	35			L
tRNA-Tyr	5423	5493	71	−1			L
COI	5495	7045	1551	1	GTG	TAA	H
tRNA-Ser2	7046	7116	71	0			L
tRNA-Asp	7118	7189	72	1			H
COII	7202	7892	691	12	ATG	T	H
tRNA-Lys	7893	7968	76	0			H
ATP8	7970	8134	165	1	ATG	TAG	H
ATP6	8128	8810	683	−7	ATG	TA	H
COIII	8811	9595	785	0	ATG	TA	H
tRNA-Gly	9596	9668	73	0			H
ND3	9669	10017	349	0	ATG	T	H
tRNA-Arg	10018	10087	70	0			H
ND4L	10088	10384	297	0	ATG	TAA	H
ND4	10378	11758	1381	−7	ATG	T	H
tRNA-His	11759	11827	69	0			H
tRNA-Ser1	11828	11896	69	0			H
tRNA-Leu1	11898	11970	73	1			H
ND5	11972	13792	1821	1	ATG	TAA	H
ND6	13789	14310	522	−4	ATG	TAA	L
tRNA-Glu	14311	14378	68	0			L
Cytb	14385	15521	1137	6	ATG	TAA	H
tRNA-Thr	15525	15597	73	3			H
tRNA-Pro	15597	15667	71	−1			L
D-loop	15668	16598	931	0			H

**Table 2:** Annotation of the mitochondrial genome of *Pethia nigrofasciata*.

Gene	Position		Size	Intergenic Nucleotides	Codon		Strand
	From	To			Start	Stop	
tRNA-Phe	1	69	69	0			H
12S rRNA	70	1025	956	0			H
tRNA-Val	1026	1097	72	0			H
16S rRNA	1098	2781	1684	0			H
tRNA-Leu2	2782	2856	75	0			H
ND1	2858	3832	975	1	ATG	TAA	H
tRNA-Ile	3845	3916	72	12			H
tRNA-Gln	3916	3986	71	−1			L
tRNA-Met	3988	4057	70	1			H
ND2	4058	5102	1045	0	ATG	T	H
tRNA-Trp	5103	5173	71	0			H
tRNA-Ala	5175	5243	69	1			L
tRNA-Asn	5245	5317	73	1			L
tRNA-Cys	5350	5416	67	32			L
tRNA-Tyr	5416	5485	70	−1			L
COI	5487	7037	1551	1	GTG	TAA	H
tRNA-Ser2	7038	7108	71	0			L
tRNA-Asp	7110	7181	72	1			H
COII	7191	7881	691	9	ATG	T	H
tRNA-Lys	7882	7958	77	0			H
ATP8	7960	8124	165	1	ATG	TAG	H
ATP6	8118	8800	683	−7	ATG	TA	H
COIII	8801	9584	784	0	ATG	T	H
tRNA-Gly	9585	9657	73	0			H
ND3	9658	10006	349	0	ATG	T	H
tRNA-Arg	10007	10076	70	0			H
ND4L	10077	10373	297	0	ATG	TAA	H
ND4	10367	11747	1381	−7	ATG	T	H
tRNA-His	11748	11816	69	0			H
tRNA-Ser1	11817	11884	68	0			H
tRNA-Leu1	11886	11958	73	1			H
ND5	11962	13785	1824	3	ATG	TAA	H
ND6	13782	14303	522	−4	ATG	TAA	L
tRNA-Glu	14304	14372	69	0			L
Cytb	14379	15519	1141	6	ATG	T	H
tRNA-Thr	15520	15591	72	0			H
tRNA-Pro	15591	15660	70	−1			L
D-loop	15661	16948	1288	0			H

**Table 3:** Nucleotide composition and skewness of *Dawkinsia filamentosa* mitochondrial genome.

Regions	Strand	Size (bp)	AT (%)	AT Skewness	GC Skewness
PCGs	H	10,872	58.1	0.139	−0.371
PCGs	L	522	57.9	−0.497	0.509
tRNAs	H	1006	56	0.130	−0.070
tRNAs	L	561	54.9	−0.136	0.233
rRNAs	H	2650	55.4	0.290	−0.107
16S rRNA	H	1695	57.4	0.289	−0.091
12S rRNA	H	955	51.8	0.293	−0.130
Full genome	H	16,598	58.1	0.166	−0.300

**Table 4:** Nucleotide composition and skewness of *Pethia nigrofasciata* mitochondrial genome.

Regions	Strand	Size (bp)	AT (%)	AT Skewness	GC Skewness
PCGs	H	10,878	60.2	0.067	−0.301
PCGs	L	522	60.2	−0.452	0.442
tRNAs	H	1003	56.9	0.103	−0.042
tRNAs	L	560	54.4	−0.121	0.224
rRNAs	H	2640	56.3	0.250	−0.082
16S rRNA	H	1684	59.1	0.260	−0.078
12S rRNA	H	956	51.7	0.231	−0.087
Full genome	H	16,948	59.9	0.108	−0.245

**Table 5:** Relative synonymous codon usage of 13 protein-coding genes of the *Dawkinsia filamentosa* mitochondrial genome.

Codon	Count	RSCU	Codon	Count	RSCU	Codon	Count	RSCU	Codon	Count	RSCU
UUU (F)	79	0.7	UCU (S)	27	0.69	UAU (Y)	49	0.84	UGU (C)	6	0.48
UUC (F)	146	1.3	UCC (S)	51	1.31	UAC (Y)	68	1.16	UGC (C)	19	1.52
UUA (L)	113	1.12	UCA (S)	98	2.51	UAA (*)	6	3.43	UGA (W)	114	1.92
UUG (L)	14	0.14	UCG (S)	5	0.13	UAG (*)	1	0.57	UGG (W)	5	0.08
CUU (L)	62	0.61	CCU (P)	18	0.34	CAU (H)	16	0.32	CGU (R)	10	0.54
CUC (L)	66	0.65	CCC (P)	34	0.64	CAC (H)	85	1.68	CGC (R)	8	0.43
CUA (L)	333	3.29	CCA (P)	159	2.97	CAA (Q)	97	1.96	CGA (R)	54	2.92
CUG (L)	20	0.2	CCG (P)	3	0.06	CAG (Q)	2	0.04	CGG (R)	2	0.11
AUU (I)	140	0.93	ACU (T)	33	0.39	AAU (N)	29	0.47	AGU (S)	11	0.28
AUC (I)	161	1.07	ACC (T)	124	1.48	AAC (N)	94	1.53	AGC (S)	42	1.08
AUA (M)	168	1.73	ACA (T)	172	2.05	AAA (K)	76	1.9	AGA (*)	0	0
AUG (M)	26	0.27	ACG (T)	6	0.07	AAG (K)	4	0.1	AGG (*)	0	0
GUU (V)	42	0.81	GCU (A)	46	0.59	GAU (D)	14	0.37	GGU (G)	29	0.48
GUC (V)	28	0.54	GCC (A)	133	1.71	GAC (D)	62	1.63	GGC (G)	38	0.63
GUA (V)	126	2.42	GCA (A)	128	1.64	GAA (E)	98	1.9	GGA (G)	145	2.39
GUG (V)	12	0.23	GCG (A)	5	0.06	GAG (E)	5	0.1	GGG (G)	31	0.51

* termination codons.

**Table 6:** Relative synonymous codon usage of 13 protein-coding genes of the *Pethia nigrofasciata* mitochondrial genome.

Codon	Count	RSCU	Codon	Count	RSCU	Codon	Count	RSCU	Codon	Count	RSCU
UUU (F)	124	1.09	UCU(S)	33	0.85	UAU (Y)	60	1.01	UGU (C)	11	0.88
UUC (F)	103	0.91	UCC(S)	42	1.08	UAC (Y)	59	0.99	UGC (C)	14	1.12
UUA (L)	176	1.73	UCA(S)	107	2.74	UAA (*)	5	3.33	UGA (W)	109	1.85
UUG (L)	9	0.09	UCG(S)	3	0.08	UAG (*)	1	0.67	UGG (W)	9	0.15
CUU (L)	92	0.9	CCU(P)	30	0.58	CAU (H)	38	0.72	CGU (R)	7	0.38
CUC (L)	65	0.64	CCC(P)	28	0.54	CAC (H)	67	1.28	CGC (R)	5	0.27
CUA (L)	243	2.38	CCA(P)	144	2.77	CAA (Q)	98	1.92	CGA (R)	58	3.14
CUG (L)	27	0.26	CCG(P)	6	0.12	CAG (Q)	4	0.08	CGG (R)	4	0.22
AUU (I)	204	1.43	ACU(T)	63	0.81	AAU (N)	46	0.8	AGU (S)	10	0.26
AUC (I)	81	0.57	ACC(T)	90	1.15	AAC (N)	69	1.2	AGC (S)	39	1
AUA (M)	165	1.66	ACA(T)	153	1.96	AAA (K)	81	1.91	AGA (*)	0	0
AUG (M)	34	0.34	ACG(T)	6	0.08	AAG (K)	4	0.09	AGG (*)	0	0
GUU (V)	55	1	GCU(A)	55	0.66	GAU (D)	18	0.47	GGU (G)	28	0.46
GUC (V)	30	0.55	GCC(A)	133	1.59	GAC (D)	58	1.53	GGC (G)	39	0.64
GUA (V)	120	2.19	GCA(A)	139	1.66	GAA (E)	91	1.77	GGA (G)	132	2.18
GUG (V)	14	0.26	GCG(A)	7	0.08	GAG (E)	12	0.23	GGG (G)	43	0.71

* termination codons.

**Table 7:** Species taxonomy used in phylogenetic analysis.

Subfamily	Species	Length (bp)	A + T (%)	GenBank Accession No.
Smiliogastrinae	*Barbodes binotatus*	16,573	57.0	KY305681
	*Barbodes semifasciolatus*	16,594	58.2	KC113209
	*Dawkinsia denisonii*	16,899	58.6	KF019637
	*Dawkinsia filamentosa*	16,598	58.1	OP595699
	*Enteromius thysi*	16,688	60.5	OP819561
	*Enteromius trimaculatus*	16,417	60.8	NC_008666
	*Hampala macrolepidota*	16,766	58.2	KF670818
	*Hampala salweenensis*	16,913	58.9	MW548258
	*Oliotius oligolepis*	16,636	58.4	ON864407
	*Oreichthys crenuchoides*	16,596	60.2	MK456608
	*Osteobrama belangeri*	16,602	60.7	KY887473
	*Pethia nigrofasciata*	16,948	59.9	OP581953
	*Pethia padamya*	16,792	58.6	ON864408
	*Pethia stoliczkana*	16,996	59.7	OP785085
	*Pethia ticto*	17,302	60.0	AB238969
	*Prolabeops melanhypopterus*	13,571	62.8	ON323520
	*Puntigrus tetrazona*	16,550	59.8	EU287909
	*Puntius sachsii*	16,587	58.2	MZ364158
	*Puntius snyderi*	16,578	59.3	KC113210
	*Sahyadria chalakkudiensis*	16,989	59.9	JX311437
	*Systomus sarana*	16,590	58.6	KU886061
Torinae	*Lepidopygopsis typus*	16,729	56.7	OR685011
Cyprininae	*Carassius auratus*	16,580	57.7	OR950752

## Data Availability

The complete mitochondrial genome sequences and annotations of *D. filamentosa* and *P. nigrofasciata* are available in the National Center for Biotechnology Information (NCBI) GenBank database https://www.ncbi.nlm.nih.gov/genbank/, accessed on 25 January 2024) under accession numbers OP595699 and OP581953, respectively.

## References

[1] Tan M., Armbruster J.W. (2018). Phylogenetic classification of extant genera of fishes of the order Cypriniformes (Teleostei: Ostariophysi). Zootaxa.

[2] Mian S., Ferdous M.J., Sarker M.Y., Islam M.J., Reza A.M., Iqbal M.M., Hossain M.A.R. (2013). Status of biodiversity and conservation of freshwater barbs in Bangladesh. World J. Fish Mar. Sci..

[3] Mohsin A.B.M., Haque S.M.M., Chaki N., Fazad F.H. (2013). Seasonal abundance of finfish in the Padma River in the Rajshahi District, Bangladesh. World J. Fish Mar. Sci..

[4] Inoue J.G., Miya M., Tsukamoto K., Nishida M. (2001). A mitogenomic perspective on basal teleostean phylogeny: Resolving higher-level relationships with longer DNA sequences. Mol. Phylogenet. Evol..

[5] Harrison R.G. (1989). Animal mitochondrial DNA as a genetic marker in population and evolutionary biology. Trends Ecol. Evol..

[6] Boore J.L. (1999). Animal mitochondrial genomes. Nucleic Acids Res..

[7] Behura S.K., Severson D.W. (2013). Codon usage bias: Causative factors, quantification methods and genome-wide patterns: With emphasis on insect genomes. Biol. Rev. Camb. Philos. Soc..

[8] Sun C.H., Sun P.Y., Lao Y.L., Wu T., Zhang Y.N., Huang Q., Zhang Q. (2023). Mitogenome of a monotypic genus, Oliotius Kottelat, 2013 (Cypriniformes: Cyprinidae): Genomic characterization and phylogenetic position. Gene.

[9] Perna N.T., Kocher T.D. (1995). Patterns of nucleotide composition at four-fold degenerate sites of animal mitochondrial genomes. J. Mol. Evol..

[10] Mullens N., Sonet G., Decru E., Virgilio M., Snoeks K., Emmanuel V. (2020). Mitogenomic characterization and systematic placement of the Congo blind barb *Caecobarbus geertsii* (Cypriniformes: Cyprinidae). Int. J. Biol. Macromol..

[11] Frank A.C., Lobry J.R. (1999). Asymmetric substitution patterns: A review of possible underlying mutational or selective mechanisms. Gene.

[12] Lazarou M., Thorburn D.R., Ryan M.T., McKenzie M. (2009). Assembly of mitochondrial complex I and defects in disease. Biochim. Biophys. Acta.

[13] Efremov R.G., Baradaran R., Sazanov L.A. (2010). The architecture of respiratory complex I. Nature.

[14] Miya M., Sato Y., Fukunaga T., Sado T., Poulsen J.Y., Sato K., Minamoto T., Yamamoto S., Yamanaka H., Araki H. (2015). MiFish, a set of universal PCR primers for metabarcoding environmental DNA from fish: Detection of more than 230 subtropical marine species. R. Soc. Open Sci..

[15] Lynch M. (1997). Mutation accumulation in nuclear, organelle and prokaryotic transfer RNA genes. Mol. Biol. Evol..

[16] Ahmed M.S., Islam N.N., Akter J.A., Sanzida N.J. (2021). DNA barcoding of Smiliogastrinae (Teleostei: Cypriniformes) of Bangladesh based on cytochrome c oxidase subunit I (coi) sequences. Zool. Ecol..

[17] Sudasinghe H., Rüber L., Meegaskumbura M. (2023). Molecular phylogeny and systematics of the South Asian freshwater-fish genus Puntius (Teleostei: Cyprinidae). Zool. Scr..

[18] Mamiatis T., Fritsch F., Sambrook J., Engel J. (1985). Molecular Cloning of a Laboratory Manual. New York, NY: Cold Spring Harbor Laboratory. Acta Biotechnol..

[19] Li R.Q., Zhu H.M., Ruan J., Qian W., Fang X., Shi Z., Li Y., Li S., Shan G., Kristiansen K. (2010). De novo assembly of human genome using massive parallel short-read sequencing. Genome Res..

[20] Zhao Q.Y., Wang Y., Kong Y.M., Luo D., Li X., Hao P. (2011). Optimizing de novo transcriptome assembly from short-read RNA-seq data: A comparative study. BMC Bioinform..

[21] Dierckxsens N., Mardulyn P., Smits G. (2017). NOVOPlasty: De novo assembly of organelle genomes from whole-genome data. Nucleic Acids Res..

[22] Lowe T.M., Chan P.P. (2016). TRNAscan-SE online: Integrating search and context for analysis of transfer RNA genes. Nucleic Acids Res..

[23] Kumar S., Nei M., Dudley J., Tamura K. (2008). MEGA: A biologist-centric software for evolutionary analysis of DNA and protein sequences. Brief. Bioinform..

[24] Chandra S., Abhilash R., Sidharthan A., Raghavan R., Dahanukar N. (2023). Complete mitogenome of *Lepidopygopsis typus*, an evolutionarily distinct, endangered cyprinid fish from the Western Ghats Biodiversity Hotspot: Phylogenetic relationships and implications for conservation. Gene.

[25] Guindon S., Gascuel O. (2003). A simple, fast, and accurate algorithm for estimating large phylogenies using maximum likelihood. Syst. Biol..

[26] Huelsenbeck J.P., Ronquist F. (2001). MBayes: Bayesian inference of phylogenetic trees. Bioinformatics.

[27] Katoh K., Misawa K., Kuma K.I., Miyata T. (2002). MAFFT: A novel method for rapid multiple sequence alignment based on fast Fourier transform. Nucleic Acids Res..

[28] Kalyaanamoorthy S., Minh B.Q., Wong T.K.F., von Haeseler A., Jermiin L.S. (2017). ModelFinder: Fast model selection for accurate phylogenetic estimates. Nat. Methods.

[29] Vrieze S.I. (2012). Model selection and psychological theory: A discussion of the differences between the Akaike information criterion (AIC) and Bayesian information criterion (BIC). Psychol. Methods.

[30] Nguyen L.T., Schmidt H.A., Von Haeseler A., Minh B.Q. (2015). IQ-TREE: A fast and effective stochastic algorithm for estimating maximum-likelihood phylogenies. Mol. Biol. Evol..

[31] Mark A.M., Terri S., Wayne P. (2013). Embedding CIPRES Science Gateway Capabilities in Phylogenetics Software Environments. XSEDE ‘13: Proceedings of the Conference on Extreme Science and Engineering Discovery Environment: Gateway to Discovery.

